# A Machine Learning Ensemble Approach Based on Random Forest and Radial Basis Function Neural Network for Risk Evaluation of Regional Flood Disaster: A Case Study of the Yangtze River Delta, China

**DOI:** 10.3390/ijerph17010049

**Published:** 2019-12-19

**Authors:** Junfei Chen, Qian Li, Huimin Wang, Menghua Deng

**Affiliations:** 1 State Key Laboratory of Hydrology-Water Resources and Hydraulic Engineering, Hohai University, Nanjing 210098, China; hmwang@hhu.edu.cn (H.W.); dengmh@hhu.edu.cn (M.D.); 2 Business School, Hohai University, Nanjing 211100, China; qianli@hhu.edu.cn

**Keywords:** urban flood, random forest (RF), RBF neural network, YRD urban agglomeration, regulation countermeasure

## Abstract

The Yangtze River Delta (YRD) is one of the most developed regions in China. This is also a flood-prone area where flood disasters are frequently experienced; the situations between the people–land nexus and the people–water nexus are very complicated. Therefore, the accurate assessment of flood risk is of great significance to regional development. The paper took the YRD urban agglomeration as the research case. The driving force, pressure, state, impact and response (DPSIR) conceptual framework was established to analyze the indexes of flood disasters. The random forest (RF) algorithm was used to screen important indexes of floods risk, and a risk assessment model based on the radial basis function (RBF) neural network was constructed to evaluate the flood risk level in this region from 2009 to 2018. The risk map showed the I-V level of flood risk in the YRD urban agglomeration from 2016 to 2018 by using the geographic information system (GIS). Further analysis indicated that the indexes such as flood season rainfall, urban impervious area ratio, gross domestic product (GDP) per square kilometer of land, water area ratio, population density and emergency rescue capacity of public administration departments have important influence on flood risk. The flood risk has been increasing in the YRD urban agglomeration during the past ten years under the urbanization background, and economic development status showed a significant positive correlation with flood risks. In addition, there were serious differences in the rising rate of flood risks and the status quo among provinces. There are still a few cities that have stabilized at a better flood-risk level through urban flood control measures from 2016 to 2018. These results were basically in line with the actual situation, which validated the effectiveness of the model. Finally, countermeasures and suggestions for reducing the urban flood risk in the YRD region were proposed, in order to provide decision support for flood control, disaster reduction and emergency management in the YRD region.

## 1. Introduction

Flood, a kind of sudden natural disaster with the characteristics of high frequency and huge loss, is closely related to other disasters, which can transfer to each other and produce a chain reaction of disasters. At present, there is no unified definition of flood [1]. It brings losses and misfortunes to people’s normal life and production activities, which are called flood disasters [2]. It has social attributes, and it is dependent on human society [3]. About two-thirds of the land area has imparity in types and degrees of flood disasters in China, and the direct economic loss caused by flood disasters accounts for about 62% of the total economic loss of various natural disasters every year [4]. Furthermore, 70% of China’s big cities and 50% of its population are located in the eastern and coastal areas. Among them, the YRD region is one of the most serious flood-prone areas, with a severe flood disaster occurring every one or two years on average [5]. Flood disasters have been on the rise in recent years and still will be a serious threat to urban sustainable development [6].

The concept of risks has been around for a long time. The risk depends on the probability of occurrence and the outcome [7]. However, the best solution to flood disasters was to control them, until the late 20th century [8,9,10]. The interaction between human society and the ecological environment has become more and more profound with the development of urbanization. The United Nations [11] raised the concept of sustainable development in the 1980s. Since then, the issue of the socio-economic ecosystem has attracted the attention of scholars at home and abroad [12,13]. In the late 1980s, the establishment of the Intergovernmental Panel on Climate Change (IPCC) marked the beginning of modern flood risk management awareness [14]. Contrary to traditional flood control theory, the modern flood risk management is a process of constantly trying to use limited resources, such as social resources, environmental resources and financial resources [15,16]. Early scholars on urban floods mainly focused on the flood disaster status in a recurrent period or a historical typical flood event and concentrated on the analysis of the flood disaster characteristics and causes [17]. For example, the floods of the Mississippi River in the United States in 1993 and the Rhine River in Europe in 1997 emphasized the need to search for better flood solutions. In the 21st century, the World Meteorological Organization published the concept document of integrated flood management for the first time, which conducted systematic studies on risk management theory, urban flood disaster, climate variability and adaptive management [18]. The flood risk analysis is the basis of flood risk management decision. After that, scholars put forward the concepts of risk, exposure and the vulnerability of flood disaster-bearing bodies systematically, forming a complete risk structure of flood disaster based on the urban background [19]. Hai et al. [20] further quantified the flood management system framework as the system of flood risk index, then developed an AHP and interval analysis method to evaluate the flood risk of Guangzhou metro system. Kotzee et al. [21] employed principal component analysis to elect key influencing factors and appraise flood elasticity index under the interaction between complex social systems and ecosystems. They further assessed the level of flood risks in the region based on the principle of various models that measure the probability and degree of floods. In this paper, driving force, pressure, state, impact and response (DPSIR) framework was proposed to analyze the influencing factors of an urban flood system, which improved the traditional PSR method that paid too much attention to environmental issues. DPSIR involves multiple subsystems, such as social, economic and ecological systems. Since changes to any element in the system lead to changes to other elements, the DPSIR framework presents this process in a complete and intuitive way. After that, the core of the research turned to in-depth analysis of data.

The constantly changing flood disaster scene has variability and unpredictability, which, in addition to the irreversibility and gray characteristics of the disaster, make it impossible for the traditional linear architecture to demonstrate the operation of this process effectively [22]. However, with the increase of the data volume and the improvement of computational accuracy, the advent of the neural network marked a turning point in flood risk assessment theory [23]. There are many kinds of neural networks, which are mainly divided into biological neural networks and artificial neural networks, among which artificial neural networks can be divided into feedforward neural networks and feedback neural networks [24,25]. The feedforward neural network has strong fitting ability and can approximate arbitrary continuous nonlinear functions. It marked the transition of artificial intelligence from a high symbolic knowledge period to a low symbolic learning period as a typical model of connectivism [26]. The backpropagation (BP) neural network is widely used in the field of disaster multiobjective algorithm and control at present. Hinton presented multiple hidden layers to replace the original single feature layer and examined the BP algorithm to train network parameters in 1985 [27,28]. Network models such as the recurrent neural network (RNN) and the convolutional neural network (CNN) are developed on this basis [29,30,31]. The deep belief neural network (DBN) is based on stacking limited Boltzmann machines and a layer of ordinary feedforward network. However, the DBN is more of a means to understand the deep learning thinking mode, rather than being practical itself [32,33]. The traditional mathematical statistical model has difficulty in deeply excavating the urban flood data. Urban flood involves the multiclassification problem of machine learning, and there is a hierarchical relationship among risk categories. The RBF network can approximate any continuous function accurately and is suitable for solving classification problems [34]. Compared with the above neural network method, the RBF network is the optimal network for input and output mapping function in a feedforward network, with fast convergence speed and high accuracy [35,36]. Many studies have verified that the RF has lower generalization error and higher prediction accuracy than other methods through a large number of theoretical and empirical studies. Lee et al. [37] practiced the decision tree, Bagging, AdaBoost and the RF methods for modeling, and the classification accuracy was obtained through 10-fold cross-validation. The analysis results showed that the RF and AdaBoost had the best accuracy. Sometimes, operation speed of the RF was much faster than AdaBoost, and there was no overfitting. The RF has a good adaptive function for many practical problems with unclear prior knowledge, nonlinear multiconstraint conditions and incomplete data [38]. As urban flood risk assessment involves a large number of indexes, it is a typical high-dimensional nonlinear problem. The RF realized the reduction of indexes by measuring the importance of indexes, thus effectively solving the high-dimensional nonlinear problem [39], in view of the fact that RBF network training index matrix did not distinguish the importance of indexes. Therefore, the random forest was introduced to establish the characteristic training weight matrix of RBF network. An urban flood risk assessment model based on RF-RBF algorithm was constructed, and the effectiveness of the algorithm was confirmed by experiments.

As one of the most mature urban agglomerations in China, the YRD region has a solid economic foundation, but the losses caused by floods have been increasing over the years [40]. This paper aims to analyze the impact indexes of flood disaster in the YRD through DPSIR framework, introduces random forest to measure the importance of impact indexes and evaluates the flood disaster level from 2009 to 2018 by RBF neural network. This paper discusses the achievements and problems of flood management in the YRD region, and proposes corresponding countermeasures. The research results can provide the basis for the regional integration of flood control and disaster reduction in the YRD, which is of great significance in improving urban flood control and management.

In summary, the ultimate goal of RF-RBF model is to alleviate urban flood disaster. The paper is organized as follows. Section 2 depicts the study area, and the YRD region was selected to evaluate flood risk levels. Section 3 describes the source of materials and research methods and presents the risk assessment model of urban flood disaster. Details on the main empirical research and assessment results are discussed in Section 4. Section 5 analyzes the research results and provides the corresponding countermeasures. The last section is the conclusion and future research needs.

## 2. Study Area

**Figure 1:**
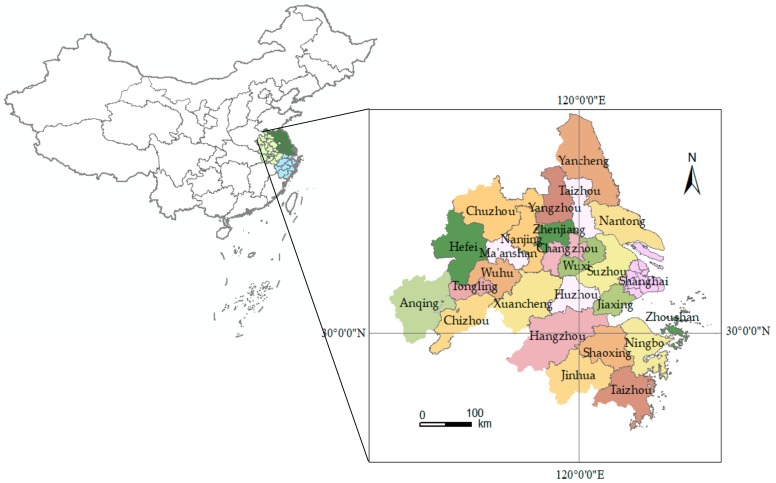
The regional schematic diagram of Yangtze River Delta (YRD) urban agglomeration.

The YRD is located in the eastern coastal region of China, which is one of the most dynamic and open regions in the economy. As an important intersection of the “One Belt and One Road” and the Yangtze River economic belt, it has a crucial strategic position in the overall opening up and modernization of the country. According to the YRD urban agglomeration development plan (2016–2020, the planning scope of the YRD urban agglomeration includes 26 cities in China: Shanghai (one municipality), Nanjing, Wuxi, Changzhou, Suzhou, Nantong, Yancheng, Yangzhou, Zhenjiang, Taizhou (nine cities in Jiangsu Province), Hangzhou, Ningbo, Jiaxing, Huzhou, Shaoxing, Jinhua, Zhoushan, Taizhou (eight cities in Zhejiang Province), Hefei, Wuhu, Ma’anshan, Tongling, Anqing, Chuzhou, Chizhou and Xuancheng (eight cities of Anhui Province), as shown in Figure 1. It covers a land area of 21.17 million square kilometers, accounting for 2.2% of the national total. Also, 11.8% of the population of China creates 21% of the GDP. The terrain of the YRD is characterized as high on all sides and low in the middle, which mostly plains. Most of the area is small watersheds with small catchment areas and storage capacity. The problem of urban floods is particularly prominent due to the influence of drainage inside the river and the water level of the rivers and lakes, coupled with the extreme rainfall events caused by typhoons in coastal areas. Here, June to September is the flood concentration period, which accounts for 80% of the flooding for the whole year. It has the characteristics of high-frequency occurrence and wide influence range of severe disasters [41]. Therefore, strengthening the risk assessment and prediction of urban agglomeration flood disasters will have a far-reaching impact on the realization of flood control and disaster mitigation, emergency management, land utilization and regional sustainable development in eastern China.

## 3. Material and Methods

### 3.1. Data Sources

This study collected the rainfall data during the flood season (June to September), socio-economic data and the data related to urban infrastructure construction of 26 cities in the YRD from 2009 to 2018. The rainfall data were gathered from the website of the National Meteorological Information Center. All the socio-economic data, such as population, GDP per square kilometer of land, urbanization rate and arable land per capita were collected through the national demographic yearbook, Shanghai statistical yearbook, Zhejiang statistical yearbook, Jiangsu statistical yearbook and Anhui statistical yearbook. The data of urban infrastructure construction, such as vegetation coverage, density of drainage networks in built-up areas and direct economic loss from flood disasters were obtained from the China urban construction statistical yearbook and the China drought and flood disaster bulletin. In addition, the water resource data were obtained from the Bureau of Hydrology and Water Resources in 26 cities. Other parts of the data were obtained through official websites or field trips by relevant departments.

### 3.2. Research Methods

#### 3.2.1. DPSIR Conceptual Framework

**Figure 2:**
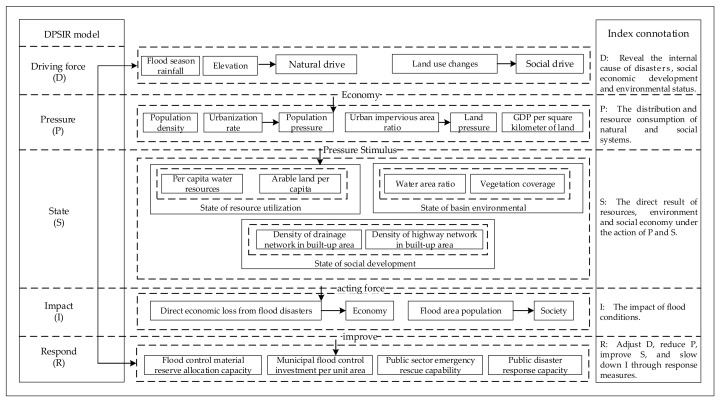
The conceptual framework of driving force, pressure, state, impact and response (DPSIR).

Urban flood disaster is a typical complex social system problem with multiple influential factors. Many factors, such as social and economic factors, environmental conditions, land planning, water and climate changes, have complex and profound influences on the probability of occurrence and consequences of urban flood disaster; the mutual influence mechanism is random and inevitable [42]. Climate changes make the analysis of these extreme events more complicated [43]. There used to be no uniform standard for quantitative research on factors affecting flood disaster, and the selection criteria of risk assessment index systems were also inconclusive [44]. To address this complexity and improve flood risk analysis methods, the organization for economic cooperation and development [45] introduced the pressure, state, response (PSR) method, then constructed a layered framework of environmental assessment indexes to describe the relationships among human activities, resources, the environment and institutional management. The early PSR framework focused excessively on environmental issues and neglected other development elements such as society, economy and institutions. There is not a one-to-one correspondence between the pressure and state, and complex social networks relationships cannot be fully revealed by a simple chain. Based on the existing problems of PSR frameworks, the European Environment Agency (EEA) first proposed the DPSIR framework when studying the relationship between environmental degradation and agriculture [46]. The DPSIR is a multifactor comprehensive conceptual evaluation model based on PSR, which is widely used in environmental system evaluation [47]. The driving force refers to the elements that directly contribute to the development of events. By separating the influence from the state, we can more intuitively understand the effect of the changing state on the “impact”. Therefore, the DPSIR framework (Figure 2) was established to analyze the causal relationship among society, economy, environment and resources in urban flood disaster system. Attempts to quantify complex problems and then implement the data-driven integration and multidimension evaluation method can provide a realistic basis for more effective evaluation results [48].

#### 3.2.2. Random Forest

**Figure 3:**
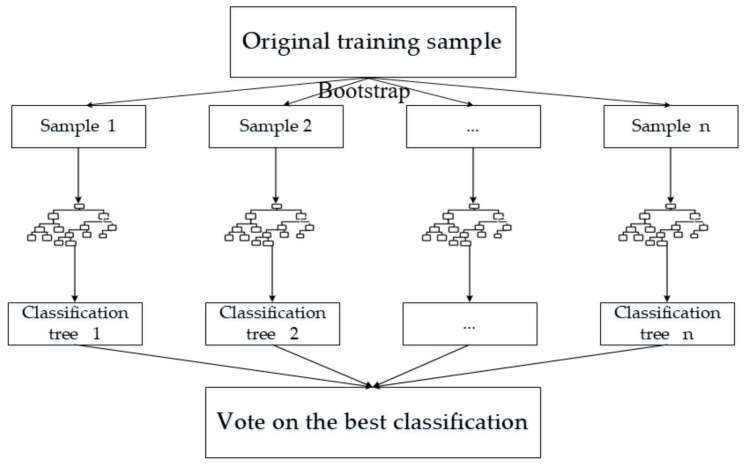
The schematic diagram of the random forest (RF) algorithm.

As we explore more sophisticated data structures, we can come up with explanations that are closer to the real world. The feature selection is the process of retaining the most effective features from the original data, and the accuracy of data classification is higher after screening [49]. At present, the selection methods of this characteristic subset mainly include the analytic hierarchy process (AHP), Pearson correlation, entropy weight method and regularization, among other algorithms. However, the RF algorithm is one of the most accurate models for classification prediction [50]. The RF is composed of a decision tree and a Bagging algorithm. As a multiclassifier algorithm based on ensemble learning, it makes full use of the concept of single classifier and sampling statistics to solve the problem of insufficient precision of a single classifier. Compared with other methods, the RF has less generalization errors and higher accuracy, which makes it suitable for solving problems without prior knowledge or with nonlinear multivariable constraints and incomplete data [51]. The steps of the RF algorithm are shown in Figure 3.

The principle of random forest is summarized as follows: In the first step, bootstrap method is used to randomly extract samples to form a training set $\left\{S_{k}\right\}$, $k\in \left\{1,2,\cdots ,N\right\}$, and the classification regression tree is generated. The second step is to randomly select the characteristics of each tree during the growth process to split the internal nodes. In this process, each tree has *M* characteristics, and *mtry* variables are randomly extracted from *M* features at each internal node. The value of *mtry* remains unchanged during the growth process of the forest. Then, the steps are repeated to maximize the growth of each tree. Finally, the randomly growing trees constitute the forest, and the new data are predicted based on the generated random forest.

Common decision tree algorithms include ID3, C4.5 and CART algorithms. The method of the ID3 algorithm is to select the best feature according to all possible values of the feature to segment the data. Specifically, the implementation step of ID3 algorithm is to calculate the information gain of each attribute separately and select the attribute with the maximum information gain for segmentation, before calculating the information entropy of the root node, as shown in Equation (1):

(1) $$Ent(D)=-\sum_{i=1}^{n}p\left(x_{i}\right)\log_{2}p\left(x_{i}\right),$$

where $p(x_{i})$ represents the probability of random event D being $x_{i}$. For example, calculate the information gain for each attribute in a feature attribute set D={M,N,⋯,W,Z} to be split. Let the set of all values of attribute d be called v; the information entropy of the v branch nodes is divided to calculate the attribute M, and the information gain of the split feature attribute M is further calculated as Gain(D,d), as shown in Equation (2):

(2) $$Gain(D,d)=Ent(D)-\sum_{v\in Value(d)}\frac{\left|D^{v}\right|}{D}Ent(D^{v}).$$

The maximum information gain is selected to divide, the analogy is carried out and finally the decision tree is obtained.

The C4.5 algorithm improves the data discretization of the ID3 algorithm by calculating the characteristics of information gain selectivity, with low accuracy, difficulty in processing missing values, complex decision tree structure and easy overfitting. The CART algorithm can be used for both regression and classification. It uses binary segmentation method to process the continuous value and makes full use of binary tree structure to segment. $t_{1}$ represents the root node, which has no inward edges but has zero or two outward edges, and $t_{i}(i=2,3,4)$ represents the inner node. In each decision tree, any leaf node is given a class label. The nonterminal node has an independent variable $X_{t}$, $X_{t}\in \left\{X_{1},\cdots ,X_{m}\right\}$, and divides different features of the record, called segment variables. As exhibited from the inner $e(T,T^{\prime })$ node within the range of T to $T^{\prime }$, it is associated with the predication of $q(T,T^{\prime })$. Among them, the $q(T,T^{\prime })$ contains only the *n*th node segmentation variable $X_{t}$. The edge of the node within a predication set $Q_{Y}$ cannot leak out; putting the edge node T in the predication of $Q_{Y}$ represents node T segmentation predication. Then, segmentation variables and the combination of predication is referred to as segmentation split criteria crit(T). The decision tree $T_{r}$ is given, and the recursive model defines the classification model, which is expressed as Equation (3):

(3) $$\begin{array}{l} c(x_{i},\cdots ,x_{m},T)=\left\{\begin{array}{l} label(T)\\ c(x_{i},\cdots ,x_{m},T_{j}) \end{array}\right.\\ D_{T_{r}}(x_{i},\cdots ,x_{m})=c\left[x_{i},\cdots ,x_{m},Root(T_{r})\right] \end{array}$$

Here, the model is expressed as label(T) when T is the leaf node, and the model is expressed as $c(x_{i},\cdots ,x_{m},T_{j})$ when T is the inner node, with $q(T,T_{j})(x_{i})=true$. $Root(T_{r})$ represents the root node of decision tree $T_{r}$.

Regression tree is actually a kind of nonparametric nonlinear regression. The CART regression has constant values on the leaf nodes and uses variance as a measure of impurity. Therefore, the measure of the CART regression tree segmentation selection is:

(4) $$\begin{array}{l} Err(T)=\frac{1}{N_{T}}\sum_{i=1}^{N_{T}}{\left(y_{i}-{\bar{y}}_{i}\right)}^{2}\\ \Delta Err(T)=Err(T)-\sum_{j=1}^{n}P\left[q_{j}(X)\left|T\right.\right]\cdot Err(T_{j}) \end{array}$$

where $N_{T}$ is the T trees of N samples in the training set.

The RF provides two feature selection methods, namely mean decrease impurity (MDI) and mean decrease accuracy (MDA). Overall, the results of MDI are consistent with MDA, but MDI is more robust than MDA [52]. Gini impurity or information gain are often used to determine the node in MDI. However, the Gini impurity represents the expected error rate of data items within the set and performs better in classification problems [53]. The smaller the Gini coefficient and the lower the Gini impurity are, the better the characteristic is. Generally, regularization and cross-validation are used to prevent model overfitting. When samples are abundant, cross-validation is a better option [54]. To clarify the characteristic selection process of the Gini coefficient, feature $M=\left[x_{1},x_{2},\cdots ,x_{c}\right]$ is assumed; if the node of feature $x_{j}$ is in the set M, the importance of $x_{j}$ in the tree i is denoted as VIM. Details are as follows:

(5) $$VIM_{ij}{}^{\left(Gini\right)}=\sum_{m\in M}VIM_{jm}^{\left(Gini\right)},$$

(6) $$Gini(t)=1-\sum_{j=1}^{k}{\left[P\left(j\left|t\right.\right)\right]}^{2},$$

(7) $$\Delta Gini(t)=(P_{L}+P_{R}-1)\sum_{j=1}^{k}p^{2}\left(j\left|t_{p}\right.\right),$$

where Gini(t) is the Gini coefficient at node t, and the smaller Gini(t) is, the more thorough the segmentation is. $P\left(j\left|t\right.\right)$ represents the conditional probability of node t at the risk level j. $t_{p}$ represents the parent node. $P_{L}$ and $P_{R}$ represent the probabilities of left and right child nodes, respectively. In general, $P_{L}+P_{R}=1$. k is the total number of categories.

The importance scores obtained from n trees were normalized, as shown in Equation (8):

(8) $$VIM_{j}{}^{\left(Gini\right)}=\sum_{i=1}^{n}VIM_{ij}{}^{\left(Gini\right)}.$$

Finally, all the obtained importance scores were normalized to obtain the results:

(9) $$VIM_{j}{}^{\prime }=\frac{VIM_{j}}{\sum_{i=1}^{c}VIM_{i}}.$$

#### 3.2.3. Radial Basis Function Neural Network

The RBF neural network is a three-layer feedforward network with a single hidden layer, which has strong approximation ability, classification ability and learning convergence rate.The set of RBF functions constructs an arbitrary basis when the input pattern vector extends to the hidden layer space, so as to transform the original problem of linear inseparability in the low-dimensional space into an approximate linear separable problem in the high-dimensional space and realize the approximation of any continuous function with arbitrary accuracy [55].

Set the ϕ is a nonlinear mapping function that maps any point x∈X in the low-dimensional space X to the high-dimensional space Y, which is termed as $\phi \left(x\right)\in Y$. If there is a function of $x_{1},\;x_{2}$ in the x space, then the inner product of Y space vector is defined as:

(10) $$<y_{1},y_{2}>=<\phi (x_{1}),\phi (x_{2})>={\left[x_{1}\right]}_{1}^{2}{\left[x_{2}\right]}_{1}^{2}+{\left[x_{1}\right]}_{2}^{2}{\left[x_{1}\right]}_{2}^{2}=k(x_{1},x_{2}),$$

where the [x] implies the sample x, the <,> is the inner product, the (,) means the sample coordinates and the k is called the kernel function. The RBF functions include the Gaussian, multiple quadratic, inverse multiple quadratic, cubic spline and thin-plate spline functions, among which Gaussian function is the most widely used [56]. In this survey, the Gaussian radial basis is used as the kernel function of the RBF neural network, and the Gaussian function is expressed as:

(11) $$k\left(x_{1},x_{2}\right)=\exp\left(-\frac{{\left\|x_{1}-x_{2}\right\|}^{2}}{2\sigma^{2}}\right),\sigma >0.$$

After x is mapped, the distance formula between this point and the origin in high-dimensional space can be expressed as:

(12) $${\left\|\phi (x_{i})\right\|}^{2}=\langle \phi (x_{i}),\phi (x_{i})\rangle =k(x_{i},x_{i})=1.$$

When the sample x is mapped to a higher dimensional space, the ϕ(x) exists on the hypersphere.

Each node of the hidden layer is just a basis in space, which is weighted by linear combination and output. This can be understood by the Weierstrass approximation theory. According to the Weierstrass approximation theorem, let $f\in C\left[a,b\right]$; then, for any ε>0 there is P such that:

(13) $$\underset{a\leq x\leq b}{\max}\left|P(x)-f(x)\right|\leq \varepsilon .$$

**Figure 4:**
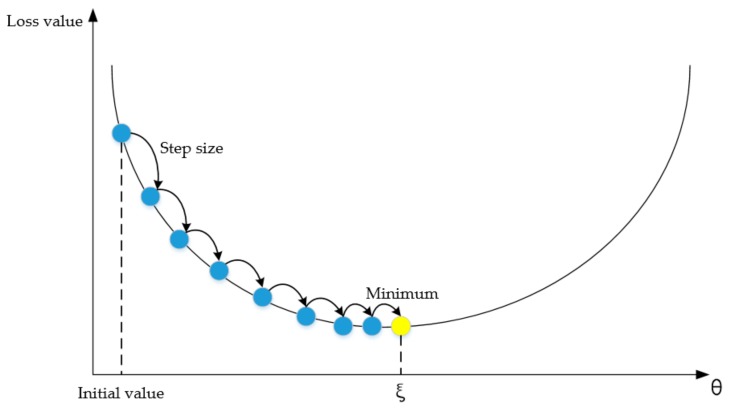
The iterative flow chart of the gradient descent method.

The key dilemma of the RBF network is the selection of the center parameters of hidden layer neurons. This is generally valued in random selection or k-means clustering method. The optimization methods include gradient descent method, Newton’s method and conjugate gradient method; the gradient descent method has the highest accuracy and is widely used. The basic function center was determined by the gradient descent method in this paper, and then the function center and distance were modified continuously, as shown in Figure 4. θ is the model parameter, and ξ is the model optimal parameter.

**Figure 5:**
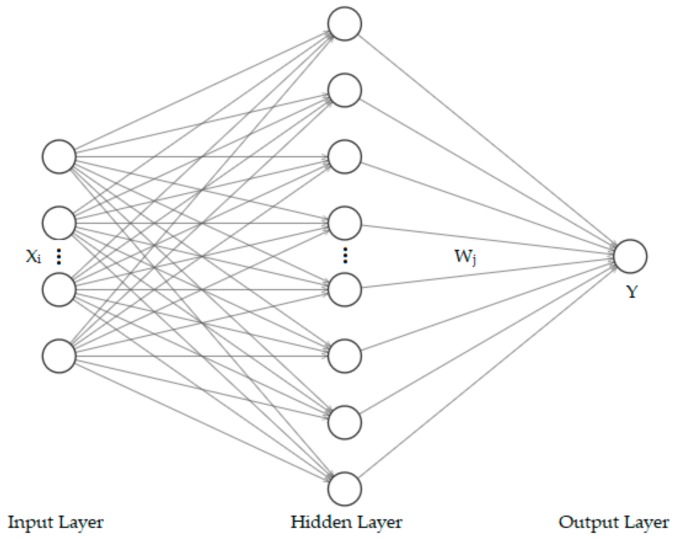
The diagram of radial basis function (RBF) structure.

As a three-layer feedforward network, the RBF neural network can transform parameters between the two layers to enable learning that can be carried out separately, and the problem of local minimization can be avoided effectively. The internal approximation principle of the network is as follows:

(14) $$y(x)=\sum_{j=1}^{M}w_{j}\phi \left(\left\|x-x_{j}\right\|\right),$$

where M represents the number of nodes in the hidden layer, and $W=\left[w_{1},w_{2},\cdots ,w_{M}\right]$ is the weight of each neural node. The hidden layer has higher dimensions in most cases, and the output layer realizes linear output through activation function. The structure of the RBF is revealed in Figure 5, including input layer, hidden layer and output layer.

DPSIR framework was adopted to dynamically acquire key elements of the urban flood system in this study, and the risk assessment model of urban flood was built based on RF-RBF. The RF was embedded to measure the importance of flood index, and the index with influence factor less than 10% was eliminated. This study creatively introduced the weight generated by RF into the RBF training network, which improved the training rate and accuracy of the model to some extent. The number of model iterations dropped from 10,000 to 3000, and the accuracy increased from 0.55 to 0.62. The results showed that the loss function was minimized when the learning rate was 0.005 and the penalty coefficient was 1. The Euclidean distance algorithm was used to fit the model. Compared with other distance algorithms, Euclidean distance ensures that the model gets the global optimal value without falling into the local optimal value [57]. The risk levels of flood disaster were classified according to the relevant standards. Finally, RBF network was employed to train the flood disaster factor matrix of the YRD urban agglomeration from 2009 to 2015, so as to verify the flood disaster risk level from 2016 to 2018. With the sensitivity to classification problems, the RBF network can realize accurate assessment of disaster risk level.

## 4. A Case Study in Yangtze River Delta (YRD)

### 4.1. System of Evaluation Index

According to the previous analysis, urban flood disaster is the result of interaction among various factors. This paper studied the regional flood characteristics of urban agglomeration in the YRD and analyzed the influencing factors of each subsystem of urban flood from the aspects of driving force, pressure, state, impact and response. On this basis, the experts in related fields were consulted [58,59]. Eighteen indexes were selected to constitute the evaluation index system of urban flood disaster (Table 1).

**Figure 6:**
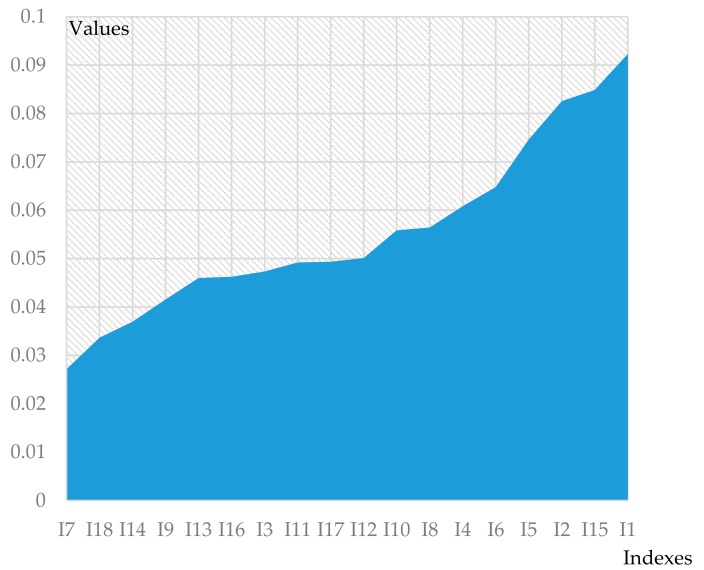
Index significance assessment results based on RF.

In order to measure the importance of various indexes to flood disaster effectively and construct a more scientific urban flood risk assessment index system, flood data of the YRD region from 2009 to 2015 (training set) were analyzed by random forest algorithm (Figure 6). Thus, the index system of urban flood was reduced by eliminating indexes with cumulative importance of less than 10%. The RF model training parameters include classification trees and node branches. As the number of trees increases, the model becomes more stable. However, when the quantity increases to a certain value, the stability improvement brought by the quantity increase will be negatively affected. After several rounds of tests, it was revealed that the optimal performance of the random model is determined when the classification tree parameter is set to 300 and the bifurcation tree with nodes parameter is set to 3.

According to Figure 6, it can be deduced that the critical indexes are rainfall, municipal flood control investment per unit area, elevation and urban impervious area ratio. On the contrary, the importance of flood area population, reserve and distribution capacity of flood control materials and per capita water resource are relatively low, accounting for less than 10%. Urban flood disaster id affected by the population and the reserve and distribution capacity of flood control materials also, which resulted in low impact on actual flood disaster risk. Therefore, these three indexes were eliminated and the top 15 indexes were employed as the input matrix of the RBF network.

### 4.2. Data Processing

Considering the particularity of urban flood disaster measurement and the difference of corresponding level values among different indexes, the data were normalized to achieve the comparability between different objectives, so as to clearly quantify the macroscopic regional distribution of urban flood disasters. Based on the selected flood indexes, the classification standard of single index of water conservancy department, the Yangtze river water resources commission and relevant literature were referred [60,61]. These index values were divided into five grades, namely, low risk (I), relatively low risk (II), medium risk (III), relatively high risk (IV) and high risk (V). The specific evaluation criteria are shown in Table 2.

### 4.3. Research Results

**Figure 7:**
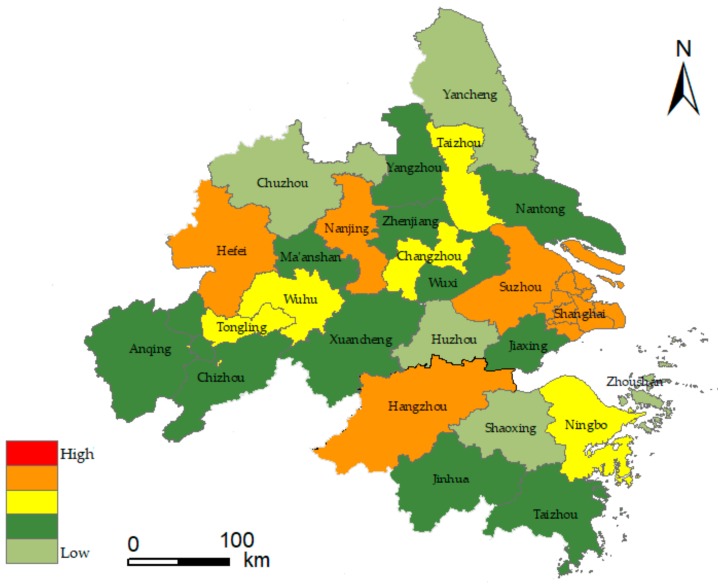
Flood risk diagram of YRD urban agglomeration in 2016.

**Figure 8:**
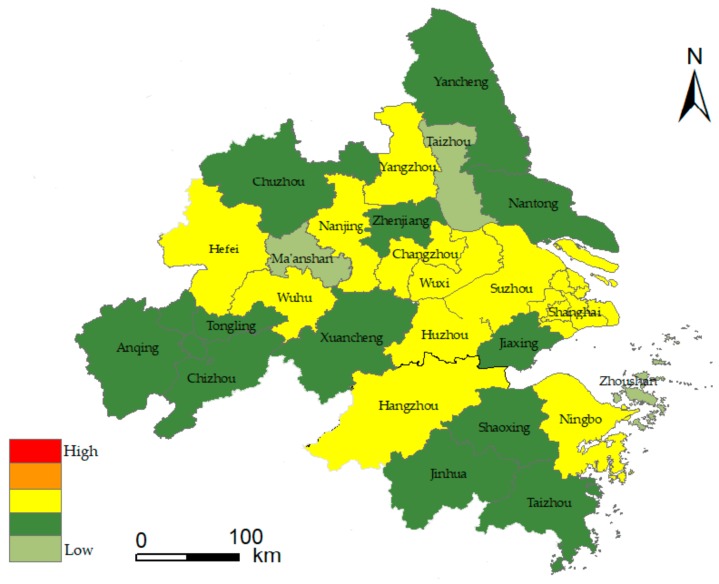
Flood risk diagram of YRD urban agglomeration in 2017.

**Figure 9:**
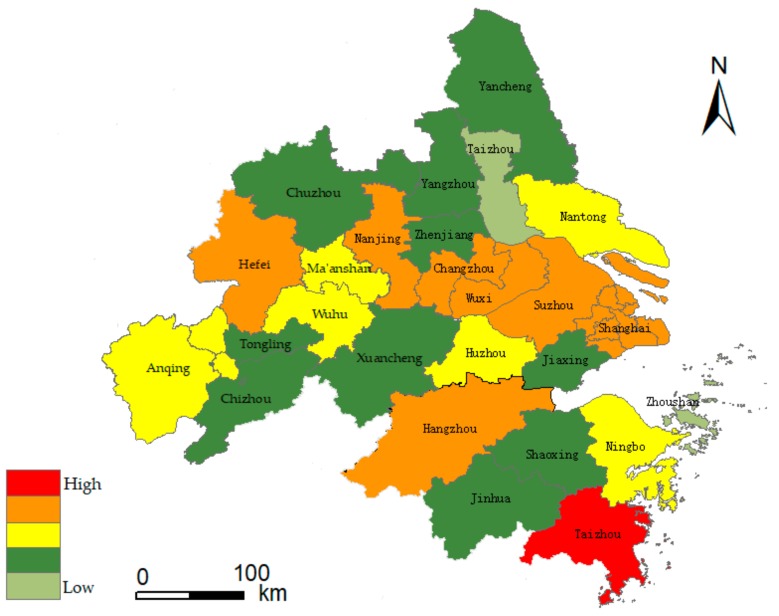
Flood risk diagram of YRD urban agglomeration in 2018.

As we all know, it is critical to divide training and testing sets properly in the field of machine learning. In general, 7:3 is known as the golden ratio, at which the model can be trained and tested well [62]. In this paper, the RBF network was constructed to train the urban flood index matrix of urban agglomeration in the YRD from 2009 to 2015. The data from 2016 to 2018 were used as test set. The model built the classifier by fitting the parameters and adjusting the parameters to the minimum value of the loss function. The test set evaluated the quality of the trained model. The RBF network used the Gaussian function to achieve the optimal approximation, and the gradient descent algorithm was used for adaptive adjustment in the training process. When the training process was iterated 10,000 times, the network loss function converged to the minimum value of 0.08, reaching the global optimal value. In order to prevent the model from overfitting, and considering the data quantity, the paper adopted the method of 5-fold cross-validation. The results showed that the prediction accuracy of the training set was 0.77, that of the verification set was 0.75 and that of the test set was 0.748. This indicated that the model has a good prediction accuracy. Finally, the flood risk assessment value of urban agglomeration in the YRD from 2016 to 2018 was predicted. Application of geographic information system software (ArcGIS) will be based on RF and RBF (RF-RBF) jointly with the risk assessment model of urban floods in the YRD region from 2016 to 2018. The risk assessment results are displayed in Figure 7, Figure 8 and Figure 9. According to the above classification, risk levels are in order of low risk, relatively low risk, medium risk, relatively high risk and high risk, corresponding to five colors on the risk diagram. Figure 7, Figure 8 and Figure 9 shows the flood risk level of urban agglomeration in the YRD from 2016 to 2018.

## 5. Discussions

### 5.1. Results Analysis

The results of flood risk evaluation are shown in Figure 7, Figure 8 and Figure 9. The YRD region from 2016 to 2018 showed a spatial pattern of high risk in the central region, low risk in the north and south regions, high risk in the east region and low risk in the west region. Among them, the risk level of urban floods is higher in economically developed areas, and the distribution of flood risk regions above level III changes from points to large areas. In the time dimension, level I flood disaster risk region decreased from 15% in 2016 to 4% in 2018. The proportion of flood risk region above level III increased from 19% in 2016 to 31% in 2018 and was greater than 42% in 2017. Some cities in the YRD have a higher level of flood risk in 2016. The characteristics of the super El Nino in 2016 led to an increase in rainfall frequency of more than 67% in the Jianghuai region of China in the summer, where the damage in Anhui province accounted for 24.04% of the total loss in the entire basin. Flood risks were relatively stable in 2017, and the region flood situation was more complex around 2018, showing a trend of more diverse levels. The flood risk in Taizhou reached level V for the first time. Apparently, flood risks have been rising over the past ten years. Furthermore, indexes such as the flood season rainfall, urban impervious area ratio, GDP per square kilometer of land, water area ratio, population density and emergency rescue capacity of public administration departments have important influence on flood risk. Therefore, flood mitigation in the YRD should focus on these aspects.

From the provincial perspective, Zhejiang and Jiangsu are both developed provinces, and the city scale of Jiangsu is generally larger than that of Zhejiang. Among them, geography is an important factor. There are more mountainous areas and less flat land in Zhejiang, which restricts the urban development to some extent. The flood risk levels are similar in Zhejiang and Jiangsu provinces, while the internal differences among cities in Jiangsu is slightly higher. The flood risk in Anhui is in the relatively low risk level, and the growth rate is relatively slow. The urbanization rate in Anhui is generally in the middle level; the city scale is relatively small, and the flood risk level is relatively low compared with other provinces. In general, the risk level of flood disaster varies among provinces, and the risk level of flood disaster shows an increasing trend in each province from 2016 to 2018.

From the perspective of cities, development results in a massive influx of population. However, the resources are limited, and urban pressure leads to an increasing risk of flood disasters. The study shows that the risk level of flood disasters is higher in provincial capitals, the municipality and developed cities. Generally, the flood risk in YRD urban agglomeration has increased significantly over the past three years due to extreme rainfall weather and low-lying terrain. This is also reflected in the research of Chau [63]. The flood risks in eastern coastal areas of the YRD, southern Jiangsu and Hangzhou Bay are relatively high, and Shanghai, Suzhou and Hangzhou are typical cities. The population size of cities such as Shanghai, Nanjing, Hangzhou, Hefei, Suzhou, Ningbo, Changzhou, Nantong, Shaoxing and Wuhu ranked among the top 11 cities in 2018, which was positively correlated with flood risk level, indicating that human disturbance factors have significant influence on urban flood risk. It is worth noting that the urban flood risk in Ningbo has remained stable in the past three years. While developing the economy, it also pays attention to the construction of urban flood control. Ningbo municipal government has fully deployed the three-year action plan of “strengthening the foundation for floods control” since 2013, and continuously strengthened the construction of urban pumping stations, with a total investment of 16 billion yuan. Since 2017, the “2020” action plan for flood control and waterlogging has been fully implemented, focusing on 21 major flood control and waterlogging projects with a planned investment of 33 billion yuan. These series of flood control measures have the propensity to prevent and control the urban flood risk effectively.

According to the urban region scale, with the rise of Nanjing metropolitan region, Hangzhou metropolitan region, Hefei metropolitan region, Suzhou–Wuxi–Changzhou metropolitan region and Ningbo metropolitan region, the flood disaster risk of urban agglomeration in the YRD region has formed a “multicenter” spatial distribution. From the flood risk diagram, the flood risk level expresses a descended distribution trend from the center area of the metropolitan to the periphery. The flood risk level in the Suzhou–Wuxi–Changzhou region of the Taihu basin has increased rapidly in a short time, which has aroused concern for the overall ecological security of this region. The urban agglomeration in Anhui section of the Yangtze River still has a large space for urbanization in the near future, and urban flood risk control is also an urgent problem to be solved in this development. Although the risk of flood factors is relatively high in the south zone of the YRD, there is no high-grade flood risk, which is consistent with the research results of Ge et al. [64].

Due to the complexity of flood disaster factors, the risk assessment level of flood disaster presents an extreme situation. For example, the typhoon Capricorn made landfall in Taizhou in 2018, and the flood disaster caused by heavy rain was relatively serious, leading to a high level of flood risk in the city. Therefore, floods are vulnerable to extreme rainfall, which is sporadic to some extent. In general, the evaluation results in this paper generally reflect the actual situation and provide a theoretical basis for the YRD urban agglomeration to prevent flood disasters more effectively and improve the ability of disaster risk management.

### 5.2. Regulation Countermeasures

The above analysis showed that the level of urban flood disaster is closely related to rainfall, topography, economic development, land use, urban flood control investment and disaster emergency response capability. The practice and experience have proved that the single concept of flood prevention can no longer adapt to the complex social environment. The concept of modern flood management is an important part of the improvement of water resources management planning and implementation within the basin, which needs to coordinate the formulation of policies and strategies at the national, river basin, provincial and sub-river basin levels [65]. The Netherlands, Mexico and other countries are actively exploring new models of multilevel integrated flood management [66,67]. The flood control should adhere to the concept of multiscenario, multidimensional and multifactor coordinated development of the society–economy–ecology. Based on the evaluation results of flood risk in the YRD region, this paper discusses how to reduce flood risk in the YRD region from three aspects: improving urban infrastructure, developing regional ecological economy and strengthening emergency response to disaster prevention and relief.

First of all, in terms of infrastructure transformation, this paper shows that the indexes such as municipal flood control investment per unit area, urban impermeable area, vegetation coverage and the density of drainage networks in built-up areas have the most significant impact on urban flood disasters. According to the characteristics of poor drainage in cities such as Shanghai and Hangzhou, governments should increase the investment in urban flood control infrastructure. A relatively perfect flood control project system should be established in cities, as well as the construction of urban rainwater pipe networks, confluence pipe networks and urban drainage pump stations. Urban wells and rainwater outlets should also be repaired. Local governments should control the growth rate of the impermeable area in Shanghai, Nanjing, Suzhou, Wuxi, Hangzhou, Ningbo and Hefei strictly, and pave the permeable urban surface. The sponge city initiative needs a joint effort of three ministries: Housing and Rural–Urban Development, Finance, and Water Resources. Urban agglomerations should establish a regional management system of the ecological environment, strengthen interprovincial coordination and protect important ecosystems such as forests, rivers, lakes and wetlands. The sponge city initiative aims to achieve this goal through permeable surfaces and “green infrastructure”, with permeable paving, waterways and wetland in urban construction. In addition, the land use pattern should be changed, and the safety management of flood detention areas and soil erosion should also be strengthened. In this way, the concept of green urbanization can be integrated into the urban construction of the YRD region.

Furthermore, in terms of regional ecological economic development, studies have shown that the key indexes include GDP per square kilometer of land, population density, urbanization rate, arable land per capita and density of highway networks in built-up areas. The government should respect the natural economic pattern and adhere to the policy of green development so that the economic development and ecological joint prevention can complement each other. The YRD city agglomeration should give full play to the central role of Shanghai, and accelerate the coordinated development of metropolitan areas in Nanjing, Hangzhou, Hefei, the Suzhou-Wuxi-Changzhou region and Ningbo, in order to optimize the spatial development pattern of “one core and five metropolitan areas”. Governments should strengthen connectivity of transport infrastructure and establish an interconnected transportation network in the YRD region. The networks of golden waterways and high-grade waterways are particularly important to enhance the radiation capacity of the YRD. For cities in Jiangsu, Zhejiang, Anhui and coastal areas that still have great potential in terms of resource carrying capacity, industry and urban space, these can be expanded appropriately, and supporting measures for satellite cities can be improved to disperse the pressure of over-rapid population growth in megacities. Cities such as Hefei, Nantong, Yangzhou, Taizhou, Shaoxing, Wuhu, Ma’anshan, Chuzhou and Xuancheng should actively develop characteristic industries, while undertaking the industrial transfer effectively. The industrial space should be distributed reasonably, and governments should adhere to the green ecological bottom-line strictly. The YRD region is vulnerable to the impact of land development pressure and soil erosion, which aggravates the frequency and loss of flood disaster. Therefore, it is necessary to accelerate the drawing of flood risk maps in the YRD region, to determine the impact of regional development strategy on the land use type in flood areas and to evaluate the loss before and after the implementation of the plan. As for the management of land use in flood areas, the overall layout of urban construction should be as far away as possible from areas with high flood risk, and the standard of flood control facilities should be improved in urban core flood areas. The government should implement zoned management of land use and increase the area of basic farmland protection areas and ecological protection areas. Moreover, the government should promote the comprehensive reform of market-based allocation of land elements and establish a cross-provincial mechanism for supplementary farmland in the YRD region. For some areas in north Jiangsu, west Anhui and west Zhejiang where the ecological environment is relatively fragile, the pace of industrial transformation and ecological restoration construction should be accelerated and the space of ecological and agricultural land should also be expanded appropriately.

Lastly, in terms of emergency response to disaster prevention and relief, the results showed that rainfall during flood season, the low-lying terrain, the emergency rescue capability of public management departments and public disaster response capability are the main factors that affect flood disasters in the YRD region. At present, nonengineering flood control measures are still not perfect. Rainfall is concentrated in the YRD in summer; in order to reduce the risk of urban floods, the three-dimensional monitoring of urban flood information should be strengthened and a real-time monitoring and rapid warning of flood information system should be developed to provide rapid response decision for urban flood emergencies. The public awareness of disaster response should be enhanced, and the disaster relief department should plan scientific personnel and material scheduling strategies to minimize the loss resulting from urban flood disasters. Relevant departments should construct an emergency management system for urban flood disasters and accelerate the establishment of the integrated emergency management mechanism in the YRD region. The YRD region should strengthen the integrated construction of flood prevention, mitigation and relief in key cities and metropolitan area. The standards for flood control and drainage of coastal cities should be improved, and the government’s policies for disaster relief and compensation should be perfected. The spatial linkage emergency plan of urban agglomeration should be established in the YRD to improve the regional capacity for flood control and disaster reduction.

## 6. Conclusions

China’s State Council approved the development plan of urban agglomeration in the YRD and the integration of YRD became a national strategy when the integration of the YRD entered a new stage in 2016. However, under the background of global warming and urbanization development, the flood disaster management in the YRD region has become increasingly urgent. This paper firstly defined the relevant concepts of urban flood disaster risk and then built the flood risk management framework from the aspect of driving force, pressure, state, impact and response. Random forest was used to screen out 15 evaluation indexes of high importance to urban flood disaster risk, such as urban impervious area, flood season rainfall, urban impervious area ratio, GDP per square kilometer of land and density of drainage networks in built-up areas. Finally, RBF neural network was used to train the flood data in the YRD from 2009 to 2015, so as to predict the flood risk level in the region from 2016 to 2018. The assessment results were presented by ArcGIS. The main research conclusions are detailed below.

Firstly, most of the existing studies on urban flood disaster risk discussed the solution of urban flood disaster from the perspective of engineering and nonengineering measures, while few of them applied machine learning theory to assess the flood disaster risk. However, the influencing factors of urban floods are multisource and complex, and the assessment of the level of urban flood risk is a multiclassification problem. The machine learning method has a good application scenario for multiclassification problems. Based on the data-driven model, this paper adopted RF to evaluate the importance of indexes, and the index weight matrix was extracted as the input of the RBF neural network. Thus, the in-depth study of samples can ensure the accuracy of the RF-RBF model, which provided great significance for flood disaster risk prediction and comprehensive management of disaster prevention and reduction in the YRD region.

Secondly, the index system of flood risk assessment was constructed from five aspects: driving force, pressure, state, impact and response. The results showed that in terms of driving force, the rainfall and terrain are the main factors affecting urban floods, which lead to the characteristics of flood and waterlogging in the YRD. In the pressure aspect, the rapid urbanization has led to the increasingly prominent contradiction between population growth and land shortage. The ratio of impermeable area of the urban underlying surface is increasing, while the water surface rate and vegetation coverage are decreasing. The urban drainage network has insufficient flood discharge capacity. In addition, the flood control capacity of small and medium-sized rivers in the basin is reduced, and the construction of flood storage and detention areas will inevitably be curtailed. As an advantageous region of grain production, the per capita cultivated land area in the YRD region only is two-thirds that of the national average. It is difficult to balance the amount of cultivated land. The issue of grain security is an alarming problem. These are the key social factors that affect urban flood disasters. As for the disaster response, the paper put forward countermeasures to reduce the flood risk in the YRD from three aspects: improving the urban infrastructure, developing regional the ecological economy and strengthening the emergency response to disaster prevention and relief.

Finally, further research could improve two potential limitations. One aspect regards the selection of indexes. The urban flood disaster is a typical complex system problem. The selected indexes still need to be perfected, considering the limitations of the current research horizon [68,69]. In the future, more scientific flood indexes will be incorporated into urban flood research. In addition, as a new research method to explain the mechanism of flood risk, the neural network model will have more new methods for the research of this problem. Based on the prospective observational studies of the flood assessment methods, we believe that these limitations will be overcome in the future.

## Figures and Tables

**Table 1:** Significance assessment of indexes based on RF model.

Index Code	Index Name	Index Weight
I1	Flood season rainfall (mm)	0.0923
I2	Elevation (m)	0.0826
I3	Urbanization rate (%)	0.0473
I4	Population density (Person/km^2^)	0.0608
I5	Urban impervious area ratio (%)	0.0746
I6	GDP per square kilometer of land (¥0.1B/km^2^)	0.0648
I7	Per capita water resources (L)	0.0272
I8	Arable land per capita (10,000/km^2^)	0.0564
I9	Water area ratio (%)	0.0416
I10	Vegetation coverage (%)	0.0559
I11	Density of highway network in built-up area (km/km^2^)	0.0492
I12	Density of drainage network in built-up area (km/km^2^)	0.0501
I13	Direct economic loss from flood disasters (¥0.1B)	0.046
I14	Flood area population (10,000)	0.037
I15	Municipal flood control investment per unit area (¥10,000)	0.0849
I16	Public disaster response capacity	0.0463
I17	Emergency rescue capacity of public administration departments	0.0494
I18	Reserve and distribution capacity of flood control materials	0.0337

**Table 2:** Classification standard of flood risk rating index.

First-Class Indicator	Second-Class Indicator	I	II	III	IV	V
Driving factor	Flood season rainfall (mm)	0–250	250–500	500–750	750–1000	1000–1250
	Elevation (m)	100–20	20–15	15–10	10–5	5–0
	Urbanization rate (%)	0–0.4	0.4–0.5	0.5–0.6	0.6–0.7	0.7–1
Pressure factor	Population density (Persons/ km^2^)	1000–1500	1500–2000	2000–2500	2500–3000	3000–5000
	Urban impervious area ratio (%)	0–0.3	0.3–0.4	0.4–0.5	0.5–0.6	0.6–1
	GDP per square kilometer of land (¥0.1B/km^2^)	0–1	1–2	2–3	3–4	4–10
State factor	Arable land per capita (10,000/km^2^)	0.5–0.2	0.2–0.15	0.15–0.1	0.1–0.05	0.05–0
	Water area ratio (%)	0.5–0.2	0.2–0.15	0.15–0.1	0.1–0.05	0.05–0
	Vegetation coverage (%)	10–6	6–5	5–4	4–2	2–0
	Density of highway network in built-up area (km/km^2^)	0–5	5–6	6–7	7–8	8–9
	Density of drainage network in built-up area (km/km^2^)	35–20	20–15	15–10	10–5	5–0
Impact factor	Direct economic loss from flood disasters (¥0.1B)	0–1.5	1.5–3	3–4.5	4.5–6	6–10
	Municipal flood control investment per unit area (¥10,000)	30–12	12–9	9–6	6–3	3–0
Response factor	Public disaster response capacity	100–85	85–80	80–75	75–70	70–0
	Emergency rescue capacity of public administration departments	100–85	85–80	80–75	75–70	70–0
